# Randomized controlled trial of intrauterine cephapirin treatment in cows of 100 days in milk or more affected by reproductive tract diseases

**DOI:** 10.3168/jdsc.2024-0691

**Published:** 2025-01-10

**Authors:** J. Dubuc, J.C. Arango-Sabogal, V. Fauteux, J. Denis-Robichaud, S. Buczinski

**Affiliations:** 1 Faculté de médecine vétérinaire, Université de Montréal, Saint-Hyacinthe, Québec, J2S 7C6, Canada; 2 Independent researcher, Amqui, Québec, G5J 2N5, Canada

## Abstract

The objective of the study was to quantify the effect of administering intrauterine cephapirin in cows ≥100 DIM affected by purulent vaginal discharge (PVD) or endometritis (ENDO) on pregnancy. A randomized controlled trial was conducted in 31 conveniently selected commercial dairy herds. In these herds, cows ≥100 DIM identified as nonpregnant during regular veterinary herd health visits were enrolled and tested for PVD (Metricheck score: fleck of pus or worse) and ENDO (cytobrush combined with a leukocyte esterase test: trace leukocytes or worse). Cows could be enrolled more than once during the study period, and the unit of interest in this study was enrollment (enrollments nested within cows nested within herds). Cows that tested positive for PVD (including cows with PVD only and those with both PVD and ENDO) or ENDO only were randomly allocated to either a treatment, which received 640 mg of intrauterine cephapirin benzathine, or a negative control that received no treatment. Cows unaffected by PVD or ENDO did not receive treatment (healthy control group). Cows from the 5 study groups were reinseminated by artificial insemination (AI) 10 d after enrollment following the same standardized timed-AI protocol (Ovsynch56). We used generalized linear mixed models (logit link) accounting for clustering at the cow and herd levels to compare study groups and compute the probability of pregnancy at AI. Season, DIM, and parity were included in the final model as confounders. Data from 1,686 enrollments (from 1,423 cows) were analyzed. A total of 498 examinations (29.6%) were classified as PVD (PVD only: n = 431; PVD and ENDO: n = 67), and 506 (29.9%) were classified as ENDO. The remaining 682 enrollments (40.5%) were classified as unaffected. Based on the final model, the probability of reproductive success was 42.9% for unaffected cows, 23.2% and 25.1% for untreated cows with PVD and ENDO, respectively, and 37.1% and 37.9% for treated cows with PVD and ENDO, respectively. These results showed that cephapirin improved the reproductive odds of cows ≥100 DIM affected by PVD or ENDO.

## Highlights

• Intrauterine treatment was assessed in cows .100 DIM with reproductive diseases.
• Treatment improved the odds of pregnancy in cows with PVD.
• Treatment improved the odds of pregnancy in cows with ENDO.
• Treated cows had similar but lower pregnancy odds than unaffected cows.

## Graphical Abstract

**Figure:**
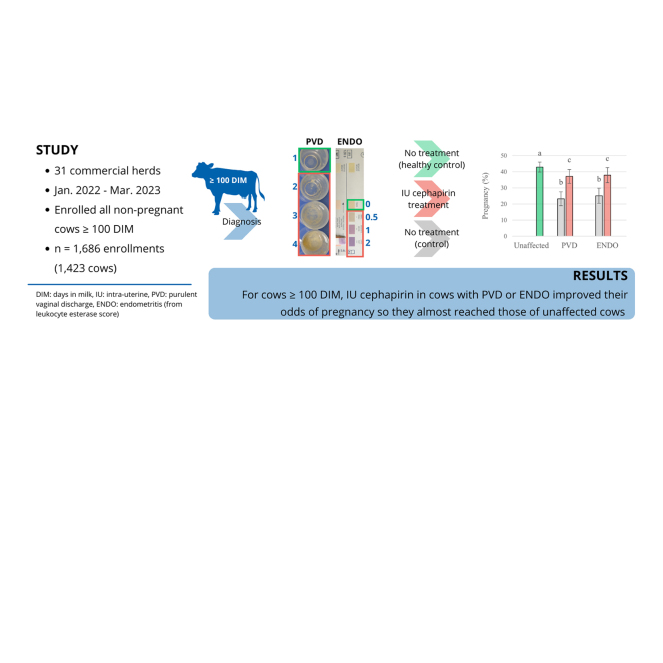
**Summary:** The objective of this study was to quantify the effect of administering intrauterine cephapirin to cows ≥100 days in milk (DIM) affected by purulent vaginal discharge (PVD) or endometritis (ENDO; from leukocyte esterase score) on pregnancy per artificial insemination. Cows were diagnosed with PVD and ENDO when being identified as nonpregnant during regular veterinary herd health visits. A randomized controlled trial was performed in 1,686 enrollments (from 1,423 Holstein cows from 31 herds) to compare cephapirin treatment to no treatment in cows with PVD or ENDO. The highest odds of reproductive success were observed in unaffected cows, but treated cows with PVD or ENDO had greater odds of pregnancy than untreated cows. Moreover, the odds of pregnancy were almost as high in treated cows as in unaffected cows.

Postpartum reproductive tract diseases like purulent vaginal discharge (**PVD**) and endometritis (**ENDO**) are common in dairy cows on commercial farms (Dubuc and Denis-Robichaud, 2017). Diagnosis of PVD and ENDO is generally performed during the postpartum period between 21 and 60 DIM (LeBlanc et al., 2002a; Dubuc et al., 2010; de Boer et al., 2014). These 2 conditions have been shown to have a detrimental effect on the subsequent reproductive performance of dairy cows during this period (LeBlanc et al., 2002b; Gilbert et al., 2005; Dubuc et al., 2010). Multiple studies have shown the effect of intrauterine cephapirin (**CEPH**; Metricure, Merck Animal Health) treatment in mitigating the detrimental effect of postpartum PVD and ENDO on reproductive performance (LeBlanc et al., 2002b; McDougall, 2003; Denis-Robichaud and Dubuc, 2015b). A study also reported that 2 CEPH treatments administered 14 d apart to treat postpartum PVD and ENDO provided better results on subsequent reproduction than only one treatment (Dubuc et al., 2021). In Canada, the use of CEPH in dairy cows is indicated for endometritis and the treatment can be repeated after 14 d.

Although some veterinarians and farmers implement systematic PVD and ENDO surveillance and treatment programs on dairy farms, our experience led us to think that many farms do not, which leaves many affected cows untreated and potentially infected during the remainder of their lactation. In such cases, undiagnosed and untreated postpartum PVD and ENDO could have detrimental effects on the reproductive performance of dairy cows ≥100 DIM. For example, repeat breeder cows (≥3 artificial inseminations [**AI**]) with ENDO were less likely to become pregnant than the one without ENDO (Bogado Pascottini et al., 2018). Before the present study, our team identified an optimal diagnostic criteria combination for PVD and ENDO in cows ≥100 DIM (Dubuc et al., 2025). Using a Metricheck device (Simcro, Hamilton, New Zealand) for diagnosing PVD and the combination of a cytobrush and a leukocyte esterase test for ENDO, we highlighted the negative impact of these diseases on the probability of pregnancy per AI (**P/AI**) in cows ≥100 DIM. Treating these affected cows represents the next logical step. However, data about the impact of CEPH on these cows are scarce. Considering the previously reported positive effect of CEPH in postpartum cows affected by PVD or ENDO (LeBlanc et al., 2002b; McDougall, 2003; Denis-Robichaud and Dubuc, 2015b), we hypothesized that CEPH will improve the P/AI in cows ≥100 DIM affected by PVD or ENDO. Thus, the study aimed to quantify the effect of administering intrauterine CEPH in cows ≥100 DIM affected by PVD or ENDO on P/AI.

A prospective randomized controlled trial was performed in 31 commercial dairy herds between January 2022 and March 2023. The study protocol was approved by the Animal Care Committee of the Université de Montréal (St-Hyacinthe, QC, Canada; 22-Rech-2061). Herds whose reproductive diseases had not been systematically assessed and treated during the postpartum period were conveniently recruited based on their location within a 1 h drive from the Bovine Ambulatory Clinic of the Université de Montréal (St-Hyacinthe, QC, Canada). Other inclusion criteria were the farmer accepting the testing of all cows ≥100 DIM identified as nonpregnant after insemination for PVD and ENDO, systematically implementing an ovulation synchronization protocol on all enrolled cows, and following it rigorously. Cows from recruited herds were systematically enrolled in the study if they were ≥100 DIM and were diagnosed nonpregnant at regular veterinary herd health visits using transrectal palpation and B-mode ultrasonography (≥29 d after last insemination). An animal health technician then examined these nonpregnant cows to diagnose PVD and ENDO. Cows that were assigned a “do not breed” status were not enrolled in this study. A cow could be enrolled in the study multiple times during the data collection period. After cleaning the vulva, a Metricheck device was used to score vaginal discharge: 0 = no discharge, 1 = clear mucus, 2 = mucus with flecks of pus, 3 = mucopurulent discharge, 4 = purulent discharge, and 5 = foul-smelling discharge (McDougall et al., 2007). A cytobrush was then used to collect a sample of the endometrium (Kasimanickam et al., 2004). A leukocyte esterase test was performed by immerging the cytobrush tip into a vial containing 1 mL of physiological saline (NaCl 0.9% Irrigation, Baxter Corporation, Mississauga, ON, Canada) and a leukocyte esterase strip (Multistix 10 SG, Bayer Corporation, Elkhart, IN) was dipped in the solution (Denis-Robichaud and Dubuc, 2015a). The colored strip was read after 2 min. Scoring was based on a colorimetric gradation of leukocytes: 0 = negative, 0.5 = trace leukocytes, 1 = small amount of leukocytes, 2 = moderate amount of leukocytes, and 3 = large amount of leukocytes (Couto et al., 2013). Both tests were performed at the farm. Cows were classified as affected by PVD if the Metricheck score was ≥2, regardless of their leukocyte esterase test result, and by ENDO if the leukocyte esterase test was ≥0.5 (Dubuc et al., 2025). Cows with PVD and ENDO were included in the PVD classification to reduce the number of study groups and allow for an achievable sample size.

Cows diagnosed with PVD or ENDO were randomly assigned (using a random number generator in Microsoft Excel [Microsoft Corp.] prepared by the research technician) to 1 of 2 groups per condition: no treatment and CEPH treatment. Therefore, 4 groups were created: (1) **PVD-NoTx**, in which cows diagnosed with PVD did not receive treatment; (2) **PVD-CEPH**, in which cows diagnosed with PVD received an intrauterine infusion of 640 mg of CEPH benzathin (Metricure, Merck Animal Health, Montréal, QC, Canada); (3) **ENDO-NoTx**, in which cows diagnosed with ENDO did not receive treatment; and (4) **ENDO-CEPH**, in which cows diagnosed with ENDO received an intrauterine infusion of 500 mg of CEPH. Further, a fifth group of cows unaffected by PVD or ENDO was also included as a healthy control. Cows in the study groups (unaffected, PVD-NoTx, PVD-CEPH, ENDO-NoTx, and ENDO-CEPH) were all assigned to the same ovulation synchronization protocol (standard Ovsynch56; Brusveen et al., 2008) and inseminated 10 d later. Pregnancy was assessed at regular veterinary herd health visits. Farmers were not allowed to breed based on heat detection (visual or activity monitor) and were requested to follow rigorously the assigned protocol. Farmers were not blind to treatment allocation because technical help (physical restraint of animals) was needed to administer CEPH.

Enrollment was the unit of interest in this study. Because cows could be enrolled more than once in the trial, unique identifiers were created by combining data on herd, cow, and DIM at enrollment. The hierarchical structure of the data was as follows: enrollments nested within cows, nested within herds. We estimated that a minimal sample of 250 enrollments per study group (unaffected, PVD-NoTx, PVD-CEPH, ENDO-NoTx, and ENDO-CEPH) was required to observe a difference between groups of at least 10 percentage points (30% vs. 40%; Denis-Robichaud and Dubuc, 2015b), accounting for cow clustering (ρ = 0.05 and average cluster size = 2), and with 95% confidence and 80% power (Dohoo et al., 2003).

Statistical analyses were computed using SAS version 9.4 (SAS Institute Inc., Cary, NC). The enrollment seasons were defined as winter (January to March), spring (April to June), summer (July to September), or fall (October to December). Descriptive statistics were computed (PROC MEANS and FREQ), and generalized linear mixed models (logit link; PROC GLIMMIX) were used to assess the effect of treatment on P/AI. These models accounted for the clustering of enrollments within cows and cows within herds (random intercepts). The study group was the exposure of interest. Because of their potential confounding effect, season, DIM, and parity were entered into all models and kept if their impact on β value was >10% (Maldonado and Greenland, 1993). Odds ratios (**OR**) and 95% CI were obtained from the final model. The predicted P/AI by study group was obtained using the LSMEANS estimated from the final model and compared (pairwise) using a Tukey-Kramer test. The predicted P/AI was also used to calculate the absolute risk modification to estimate how many cows needed treatment to obtain one additional pregnancy using the number needed to treat formula (Chatellier et al., 1996). Sensitivity analyses were conducted on datasets excluding cows positive to PVD and ENDO, and excluding repeated enrollments within the same cows.

Recruited herds had 40 to 350 milking cows (median = 85), were housed in tiestall (n = 24; 77.4%) or freestall (n = 7; 22.6%) barns and were TMR-fed (n = 19; 61.3%) or component-fed (n = 12; 38.7%). In the recruited herds, first insemination was done following an Ovsynch (n = 7) or a Double-Ovsynch (n = 24) protocol, with first insemination after 60 DIM. Of the 1,718 initial enrollments, 32 (all different cows representing 1.9% of enrollments) were excluded because of the farmer's lack of compliance with the study protocol (19 cows were never inseminated after enrollment, and 13 were not inseminated based on the assigned ovulation synchronization protocol). A total of 1,686 enrollments from 1,423 Holstein cows in 31 commercial dairy herds were used for the analyses. Most cows were enrolled once (n = 1,244 cows), but some cows were enrolled up to 4 times. Of the 179 cows with multiple enrollments, 43 cows received no treatment, 90 cows received 1, 44 cows (24.6%) received 2, 2 received 3, and none received 4. Overall, 498 enrollments (29.6%) were categorized as PVD (PVD only: n = 431, PVD, and ENDO: n = 67), and 506 (29.9%) as ENDO. At the herd level, the median (minimum–quartile 1–quartile 3–maximum) proportion of positive events was 26.9% (2.5–17.8–31.1–38.1) for PVD and 33.4% (4.5–27.8–41.5–77.3) for ENDO. The overall P/AI for enrolled cows was 35.7% (n = 602), with a herd level median of 36.1% (19.5–30.1–43.2–49.7). The proportions of enrollments stratified by season were 32.1% (n = 541) for winter, 21.3% (n = 359) for spring, 22.8% (n = 384) for summer, and 23.8% (n = 402) for fall. The distributions of parity, DIM, and number of inseminations before enrollment were similar among the study groups (Table 1). The study group, DIM at enrollment, season, and parity were retained in the final model. All affected cows had lower odds of pregnancy than unaffected cows. Among the affected cows, however, the odds of pregnancy were greater in treated cows (the referent is unaffected cows; PVD-CEPH: OR = 0.86, 95% CI = 0.74–0.97; ENDO-CEPH: OR = 0.88, 95% CI = 0.77–0.98) than in untreated cows (the referent is unaffected cows; PVD-NoTx: OR = 0.54, 95% CI = 0.43–0.66; ENDO-NoTx: OR = 0.59, 95% CI = 0.47–0.71). Results were similar, with slightly wider 95% CI, when cows positive to PVD and ENDO or when multiple enrollments were removed (Table 2). The predicted probabilities from the final model suggested that cows with PVD had a 14 percentage points higher P/AI when treated compared with untreated animals, and cows with ENDO had a 13 percentage points higher P/AI when treated compared with untreated animals (Figure 1). Using this result, the number of cows that needed to be treated to obtain one additional pregnancy was 7 for PVD and 8 for ENDO. Moreover, treated cows had P/AI (PVD-CEPH = 37%; ENDO-CEPH = 38%) almost as high as unaffected cows (unaffected = 43%).

**Table 1:** Descriptive statistics of 1,686 enrollments (≥100 DIM) from 1,423 cows from 31 commercial herds enrolled in a randomized controlled trial quantifying the effect of intrauterine cephapirin (CEPH) as a treatment for purulent vaginal discharge (PVD) and endometritis (ENDO)^1^

Item	n (%)	DIM at enrollment			Parity			Inseminations prior to enrollment		
		Min	Med	Max	Min	Med	Max	Min	Med	Max
All		100	173	427	1	3	10	1	1	4
Unaffected^2^	682 (40.5)	100	168	401	1	3	10	1	1	4
PVD-NoTx^3^	249 (14.8)	102	169	421	1	3	9	1	1	4
PVD-CEPH^4^	249 (14.8)	105	175	427	1	3	9	1	1	4
ENDO-NoTx^5^	252 (14.9)	108	174	419	1	3	10	1	1	4
ENDO-CEPH^6^	254 (15.0)	101	172	434	1	3	9	1	1	4

1 Min = minimum; Med = median; Max = maximum.

2 Unaffected: negative for PVD and ENDO.

3 PVD-NoTx: positive for PVD (PVD only: n = 214; PVD and ENDO: n = 35) and did not receive CEPH treatment.

4 PVD-CEPH: positive for PVD (PVD only: n = 217; PVD and ENDO: n = 32) and received CEPH treatment.

5 ENDO-NoTx: positive for ENDO and did not receive CEPH treatment.

6 ENDO-CEPH: positive for ENDO and received CEPH treatment.

**Table 2:** Sensitivity analyses for the generalized linear mixed model of the effect of purulent vaginal discharge and endometritis, and intrauterine cephapirin on pregnancy per artificial insemination^1^

Item	All	Exclusion of PVD and ENDO cows	Exclusion of multiple events
n events (n cows)	1,686 (1,423)	1,619 (1,370)	1,423 (1,423)
Unaffected cows	Referent	Referent	Referent
PVD-NoTx	0.54 (0.43–0.66)	0.55 (0.41–0.68)	0.55 (0.43–0.69)
PVD-CEPH	0.86 (0.74–0.97)	0.88 (0.75–0.98)	0.88 (0.72–0.99)
ENDO-NoTx	0.59 (0.47–0.71)	0.60 (0.48–0.72)	0.58 (0.45–0.73)
ENDO-CEPH	0.88 (0.77–0.98)	0.87 (0.75–0.99)	0.88 (0.76–0.99)

1 Data are presented as odds ratio (95% CI). PVD = purulent vaginal discharge; ENDO = endometritis; NoTx = no treatment; CEPH = intrauterine cephapirin.

**Figure 1:**
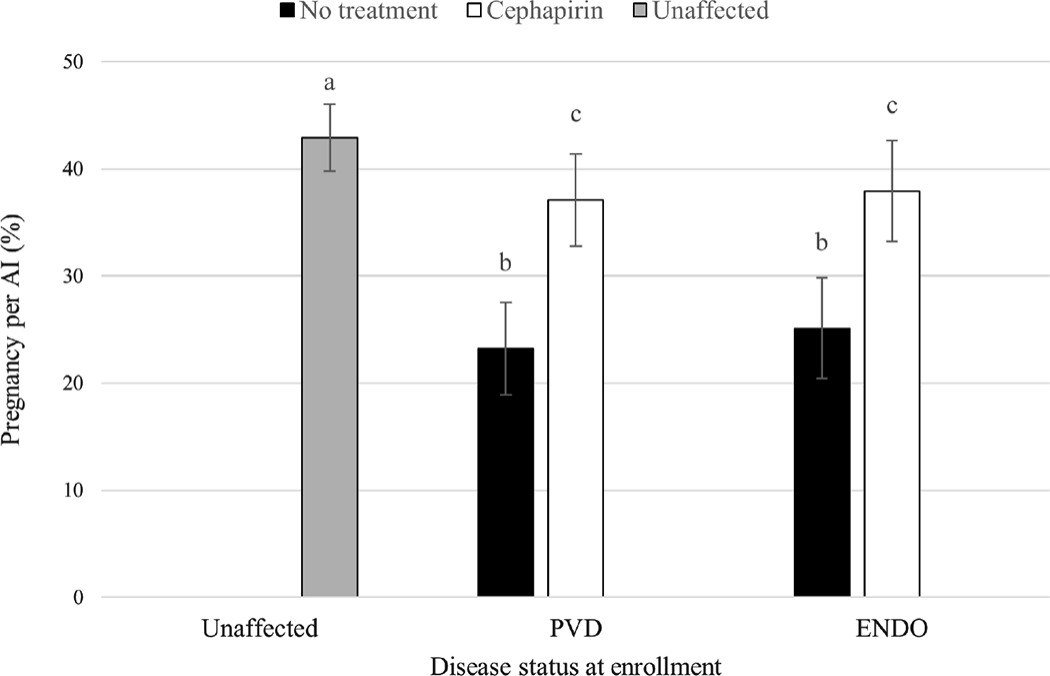
Predicted probability (LSM ± SEM) of reproductive success at subsequent insemination (P/AI) in cows ≥100 DIM enrolled in a randomized controlled trial assessing the effect of intrauterine cephapirin on the P/AI of cows with purulent vaginal discharge (PVD) and endometritis (ENDO). Different letters indicate a significant difference (*P* < 0.05; Tukey-Kramer).

There are no other comparable studies on cows ≥100 DIM, but the magnitude of this effect is similar to what has been previously reported on cows during the postpartum period (LeBlanc et al., 2002b; Denis-Robichaud and Dubuc, 2015b; Dubuc et al., 2021). Our results highlight the benefits of treating cows with PVD or ENDO with intrauterine CEPH later in lactation than in previous studies. They also suggest the usefulness of implementing on-farm surveillance strategies for PVD and ENDO, even after 100 DIM, for improving the P/AI of cows. From a herd health surveillance perspective, diagnosing and treating cows for PVD and ENDO during the postpartum period is ideal to mitigate their negative reproductive impact at first and subsequent inseminations. In situations where such postpartum surveillance cannot be established on farms, our findings suggest that identifying these conditions in cows ≥100 DIM and treating them has the potential to also improve the reproductive performance of cows on dairy farms.

Although PVD and ENDO have been used to identify cows that would benefit from intrauterine CEPH treatment, they do not give information about the presence of bacteria in the reproductive tract or about their susceptibility to CEPH. This is a limitation that should be considered by veterinarians for prudent use of antibiotics. Additionally, the bacteria present in the reproductive tract of nonpregnant cows ≥100 DIM, and their susceptibility to CEPH, should be described by future research to better support the use of CEPH in this population. A more adequate tool to identify cows with susceptible infection would also be beneficial toward prudent antimicrobial use, but this was beyond the scope of the present study. During this study, the use of intrauterine CEPH in dairy cows was legal for veterinarians in Canada, and although susceptibility testing on isolated bacteria is ideal, it was not mandatory. This is likely not the case in all jurisdictions and our findings must be interpreted accordingly.

Future studies could also assess the impact of treatment repetition. In the present study, only a limited proportion of enrollments were repeated enrollments for the same cows, for which they could receive CEPH or NoTx on each repetition. The effect of treatment repetition on the odds of pregnancy was not assessed because the sample size and study design were not adequate for this objective. However, repeated enrollments were addressed in our analyses to minimize biases related to nonindependence and reduce type 1 error. Moreover, our sensitivity analyses suggest similar results when including only the first enrollment of each cow.

When looking at our results, one should keep in mind that the proportion of cows testing positive for PVD or ENDO in the present study may not reflect the reality on commercial farms. The occurrence of PVD and ENDO in cows ≥100 DIM and their variability between herds remain unclear. The distribution in the present study resembled that in the study we conducted to identify the diagnostic thresholds (Dubuc et al., 2025). Indeed, PVD in these herds varied from 4.5% to 37.4% (median = 18.6%), whereas ENDO varied from 9.1% to 78.7% (median = 38.2%). It could be assumed that herds subject to a systematic postpartum surveillance and treatment program would likely have a lower proportion of PVD- and ENDO-positive cows after 100 DIM than herds without such a program, but this remains to be explored. It should also be remembered that enrollments positive for both PVD and ENDO were assigned to the PVD groups (NoTx or CEPH) for practical reasons. It has been documented that cows can have both PVD and ENDO and that the individual detrimental effect of each disease is additive (Dubuc et al., 2010). Therefore, it would have been ideal to have randomized treatment allocation groups within this specific population. However, reaching a sufficient sample size during data collection was logistically unfeasible. We included cows with both PVD and ENDO in the same group as the diagnostic test for PVD is easy to use, but our findings could have been biased by including cows with both conditions. When we assessed the effectiveness of CEPH on P/AI in PVD cows only (by excluding cows with both PVD and ENDO from data analysis), we found a similar improvement in treated cows. Moreover, it was suggested that the presence of PVD and ENDO were manifestations of the same reproductive tract condition (Wagener et al., 2017), which was supported by the similar improvement following treatment found in the present study.

In conclusion, treating cows ≥100 DIM affected by PVD or ENDO with CEPH was beneficial for improving their P/AI. When comparing treated and untreated cows, there was a 14 point P/AI increase for cows classified as PVD and a 13 point increase for cows classified as ENDO. Moreover, the pregnancy odds of treated cows were close to those of unaffected cows.
